# Spatial analysis of cardiovascular mortality and associated factors around the world

**DOI:** 10.1186/s12889-022-13955-7

**Published:** 2022-08-16

**Authors:** Emerson Augusto Baptista, Bernardo Lanza Queiroz

**Affiliations:** 1 Center for Demographic, Urban and Environmental Studies, El Colegio de México A.C., 14110 Mexico City, Mexico; 2 Universidade Federal de Minas Gerais / Cedeplar, Belo Horizonte, 31270-901 Brazil

**Keywords:** Mortality, Cardiovascular mortality, Spatial analysis, Associated factors, Spatially autoregressive models

## Abstract

**Background:**

Cardiovascular disease (CVD) is one of the most serious health issues and the leading cause of death worldwide in both developed and developing countries. The risk factors for CVD include demographic, socioeconomic, behavioral, environmental, and physiological factors. However, the spatial distribution of these risk factors, as well as CVD mortality, are not uniformly distributed across countries. Therefore, the goal of this study is to compare and evaluate some models commonly used in mortality and health studies to investigate whether the CVD mortality rates in the adult population (over 30 years of age) of a country are associated with the characteristics of surrounding countries from 2013 to 2017.

**Methods:**

We present the spatial distribution of the age-standardized crude mortality rate from cardiovascular disease, as well as conduct an exploratory data analysis (EDA) to obtain a basic understanding of the behavior of the variables of interest. Then, we apply the ordinary least squares (OLS) to the country level dataset. As OLS does not take into account the spatial dependence of the data, we apply two spatial modelling techniques, that is, spatial lag and spatial error models.

**Results:**

Our empirical findings show that the relationship between CVD and income, as well as other socioeconomic variables, are important. In addition, we highlight the importance of understanding how changes in individual behavior across different countries might affect future trends in CVD mortality, especially related to smoking and dietary behaviors.

**Conclusions:**

We argue that this study provides useful clues for policymakers establishing effective public health planning and measures for the prevention of deaths from cardiovascular disease. The reduction of CVD mortality can positively impact GDP growth because increasing life expectancy enables people to contribute to the economy of the country and its regions for longer.

**Supplementary Information:**

The online version contains supplementary material available at 10.1186/s12889-022-13955-7.

## Background

The toll of non-communicable diseases (NCDs) is very large, making them the leading cause of death globally, and one of the major health challenges of this century in both developed and developing countries [1–3]. In 2017, approximately 73% (41 million) of the 55 million deaths that occurred in the world were due to NCDs. The major NCD responsible for these deaths are cardiovascular diseases (CVDs), accounting for 17.8 million deaths, or 31.8% of all global deaths. These numbers also represent a 49% increase in deaths from CVDs compared to 1990 [4].

The World Health Organization (WHO) [5] estimates that over three quarters of CVD deaths take place in low- and middle-income countries, where exposure to risk factors associated with CVD mortality still persists, although efforts are underway to minimize its impacts on public health. These concerns and the importance of a reduction in CVD mortality are shared and recognized in the third Sustainable Development Goal (SGD) [6]. However, most studies relating to CVD mortality and its impact are concentrated on developed countries [7–9], with several focusing on some, especially the United States [3, 10].

The risk factors for CVD include demographic (such as population ageing), socioeconomic (education, income, and poverty), behavioral (tobacco use, a sedentary lifestyle, and an unhealthy diet), environmental (the exposure to poor air quality), and physiological (high blood pressure and high blood cholesterol) factors [11, 12]. There are also several underlying determinants and drivers, such as urbanization and hereditary factors [5]. However, the spatial distribution of these risk factors, as well as CVD mortality, are not uniformly distributed across countries. In this paper, we make extensive use of CVD mortality estimates from the Global Burden of Disease to investigate the global pattern of mortality and associated factors. We hypothesize that the spatial spillover process, if any, may be relevant in understanding the role of risk factors in CVD mortality disparities, that is, that the relationship between them is consistent across space and operate similarly in adjacent countries.

Lopez an Adair [8] found that the decline in mortality rates by cardiovascular diseases has slowed down in recent years and, in some countries, estimates it is even an increase in rates. They suggest several possible explanations for the change, since they are occurring across different contexts. Roth et al. [13] describe persistent differences across gender, with males having higher mortality than females, and an increasing risk of mortality by CVD in less developed economies related to changes in population age structure and overall socioeconomic conditions [14, 15]. Roth et al. [13] further argues that a myriad of factors explain recent trends in CVD mortality and indicates a large variation across and within regions of the world. Gu et al. [16] show that higher income per capita was associated with lower mortality rates by cardiovascular disease in Eastern and Southeastern Asian countries. The results also indicated that the association between the variables tends to decline as the income level reaches a certain level. Mehta et al. [17] show that the slowed in the progress of life expectancy in the United States is explained by increased in CVD mortality. In addition, they point out that the increase in CVD mortality can be explained by increasing obesity levels and high prevalence of diabetes. However, most of the studies focused on specific countries or in a group of more developed countries. There are still few studies looking at the global trends and impacts of cardiovascular disease mortality, especially on how low- and middle-income countries are situated.

The goal of this study is to compare and evaluate some models commonly used in mortality and health studies to investigate whether the CVD mortality rates in the adult population (over 30 years of age) of a country are associated with the characteristics of surrounding countries from 2013 to 2017. This is an attempt to advance and elucidate some issues (spatial, demographic, socioeconomic, behavioral, and epidemiological) related to the main cause of death in the world.

## Data and methods

### Study design and level of analysis

The Global Burden of Disease Study 2017 [4], coordinated by the Institute for Health Metrics and Evaluation (IHME) and publicly available online (http://www.healthdata.org/), was created to provide comprehensive and comparable global health metrics. Estimates of cause-specific mortality, burden of diseases, injuries, and risk factors are reported by year (1990–2017), location, age, and sex. IHME uses data from 1,257 census and 761 population registry location-years to produce these estimates for 195 countries and territories. In this study, we concentrate on 187 countries and territories. This difference occurs because in these 8 countries or territories the data of the explanatory variables used in this study are not available, either because they are countries with an uncertain “political” definition, such as Taiwan, or because they are considered territories of other countries, such as American Samoa, Guam, Northern Mariana Islands, Puerto Rico, and Virgin Islands, all United States territories. The list containing 187 countries or territories is in Additional file 1.

The IHME’s model used to build these estimates already has a spatial component, and this could affect our results. However, Foreman et al. [18] show that the methodology uses a value of ζ = 0.9 for countries with data. This implies that 90% of the weight in the local regression is given to observations from the same country. Another 9% of the weight comes from data from the same region, but outside the country, and without specifying a neighborhood relationship. Lastly, only 1% is given to data in other parts of the super-region. In other words, the estimates do not have a great spatial influence, at least at the country level, since the model gives much greater weight to the country (90%) and only residual the region.

Finally, for purposes of analysis, and in order to adjust the annual fluctuations that may occur, we use one 5-year period (2013–2017). Deaths from cardiovascular disease (*n* = 83,999,570, that is, annual average of 16,799,914) and population were organized by age (in 5-year age groups up to 95 years or more). We then calculate age-standardized death rates per 100,000 for each country using the world population in 2010 as the standard. All calculations and routines presented in this paper were performed in *R* (basic statistics) and *Geoda* (spatial statistics) software.

### Variables and data source

This study assembles data from multiple sources. The country-level age-standardized crude mortality rate from cardiovascular disease (CMRCVD) is the dependent variable of this study. Data on this cause-specific, as well as age-specific (population over 30 years and in 5-year age groups up to 95 years or more), come from the Global Burden of Disease Study 2017 [4].

We obtained the gross domestic product per capita (GDP per capita) and the expected years of schooling from the United Nations Development Programme (UNDP) [19]. The first is measured in purchasing power parity (2011 PPP $). This is one of the most widely used socioeconomic predictors of mortality / health, and this relationship has been widely discussed in the literature [9, 16, 20–26]. The second refers to the number of years of schooling that a child of school entrance age can expect to obtain if prevailing patterns of age-specific enrolment rates persist throughout the child’s life. A vast literature has persistently shown the inverse association between educational attainment and mortality / health, almost all indicating that individuals with better education are healthier and live longer [16, 27–32]. Both data are from 2015, which is equivalent to the middle of the period used in this study (2013–2017).

Annual percentage of population at mid-year (2015) residing in urban areas was obtained from the United Nations Department of Economic and Social Affairs (UNDESA) [33]. Urbanization is an important factor in CVD mortality, as it changes the behavior of individuals to a sedentary lifestyle, a diet rich in salt intake, sugar, and fat, and tobacco addiction. Add to this, the problem of criminality and a loss of the traditional social support mechanisms [7, 16, 34–36].

Lastly, the variable cigarette use comes from Institute for Health Metrics and Evaluation (IHME) [37]. This is an estimate of the prevalence of daily smoking in 2012 (most recent data), that is, the percentage of men and women, of all ages, who smoke daily. In this work, the data are aggregated at the country level, in other words, are country-related features. It has been well established in the literature that smoking is an important risk factor for certain types of diseases, especially for chronic non-communicable diseases (NCDs), such as cancers and cardiovascular diseases [38–43].

### Spatially autoregressive models

As the general choice for analyzing non-spatial data, at the same time it is the starting point for all spatial regression studies, Ordinary Least Squares (OLS) is a classic linear regression model that estimates the linear relationship between the dependent variable and the explanatory variables. This model is applied regularly in ecological demographic research and captures the average strength and significance of the explanatory variables, but assumes that the relationship between the dependent and independent variables in each location is equally weighted over all data. In other words, it presupposes that the dependent variable (CMRCVD) in a country *i* are independent of rates in neighboring country *j* and that the residuals of the model are normally distributed and that they have constant error variance [44–46]. In this study, we specify the OLS model as:

1 $$CMRCVD = {\beta }_{0} + {\beta }_{1}*GDP per {capita + \beta }_{2}*\% {urbanization + \beta }_{3}{*Schooling + \beta }_{4}*Cigarettes + \varepsilon$$

where CMRCVD is the dependent variable, GDP per capita, % urbanization, schooling and cigarettes are the explanatory variables, the βs are regression coefficients, and $\varepsilon$ is error term.

When spatial data are considered, however, that is, when a value in one location depends on the values of its neighbors, the OLS regression model presents a series of problems, such as the errors are no longer uncorrelated (autocorrelation) and may not be normally distributed, heteroskedasticity (non-constant variance) of the model residuals, and non-stationarity of the distributional parameters. These problems are usually seen as various representations of spatial structure within the data [44], which leads us to adopt a spatial model.

Several spatial model specifications can be observed in the literature, but two are the most commonly used: spatial lag model and spatial error model. Both are spatially autoregressive models, with the first adding a spatially lagged dependent variable $Wy$ to the conventional regression formula (Eq. 2) and the second modeling the spatial dependence among the error term (Eq. 3) [47, 48].

2 $${y}_{i} = { \rho {W}_{i}{y}_{i}+ \beta X}_{i}+{u}_{i}$$

where $y$ is a $n\times 1$ vector of observations on the dependent variable (CMRCVD), $\rho$ is spatial autoregressive parameter, ${W}_{i}{y}_{i}$ is the spatially lagged dependent variable for weights matrix $W$ with a $n\times n$ spatial lag operator, $X$ is an $n\times k$ matrix of observations on the explanatory variables with $k\times 1$ coefficient vector $\beta$, and ${u}_{i}$ is a vector of error terms.

3 $${y}_{i} = { \beta X}_{i}+{\lambda {W}_{i}{\varepsilon }_{i}+ u}_{i}$$

where $X$ is an $n\times k$ matrix of observations on the explanatory variables with $k\times 1$ coefficient vector $\beta$, $\lambda$ is spatial autoregressive parameter, $\varepsilon$ is error term weighted by the weight matrix $W$, and ${u}_{i}$ is the random error (not explained by the model).

Following this approach, several studies on mortality and health have applied the two spatially autoregressive models and showed the importance of considering location in the analyzes [44, 49–53].

## Analytical strategy

This study will first present the spatial distribution of the age-standardized crude mortality rate from cardiovascular disease, as well as will conduct an exploratory data analysis (EDA) to obtain a basic understanding of the behavior of the variables of interest. We will then apply the ordinary least squares (OLS) to the country level dataset. As OLS does not take into account the spatial dependence of the data, we will apply two spatial modeling techniques, that is, spatial lag and spatial error models. Finally, we will compare the regression results of the three models in terms of Akaike Information Criterion (AIC), log likelihood, and R^2^, on which the performance of the models can be assessed. It is worth mentioning that, although we have presented the values of R^2^ for the models, it is not possible to make a direct comparison between an usual R^2^ (OLS model) and a *pseudo*-R^2^ (spatial models). While the first can be interpreted as an indication of the proportion of explained variance by the model, *pseudo*-R^2^, which is the squared correlation between the observed and predicted values, is only a rough indicator of relative fit and can be used as a rough guideline in model selection [45]. Lastly, this study will employ the Queen (first-order) adjacency weights matrix. This criterion correlates countries with their neighbors, regardless of their direction, to define whether they are neighbors or not.

## Results

### Exploratory analysis

**Fig. 1:**
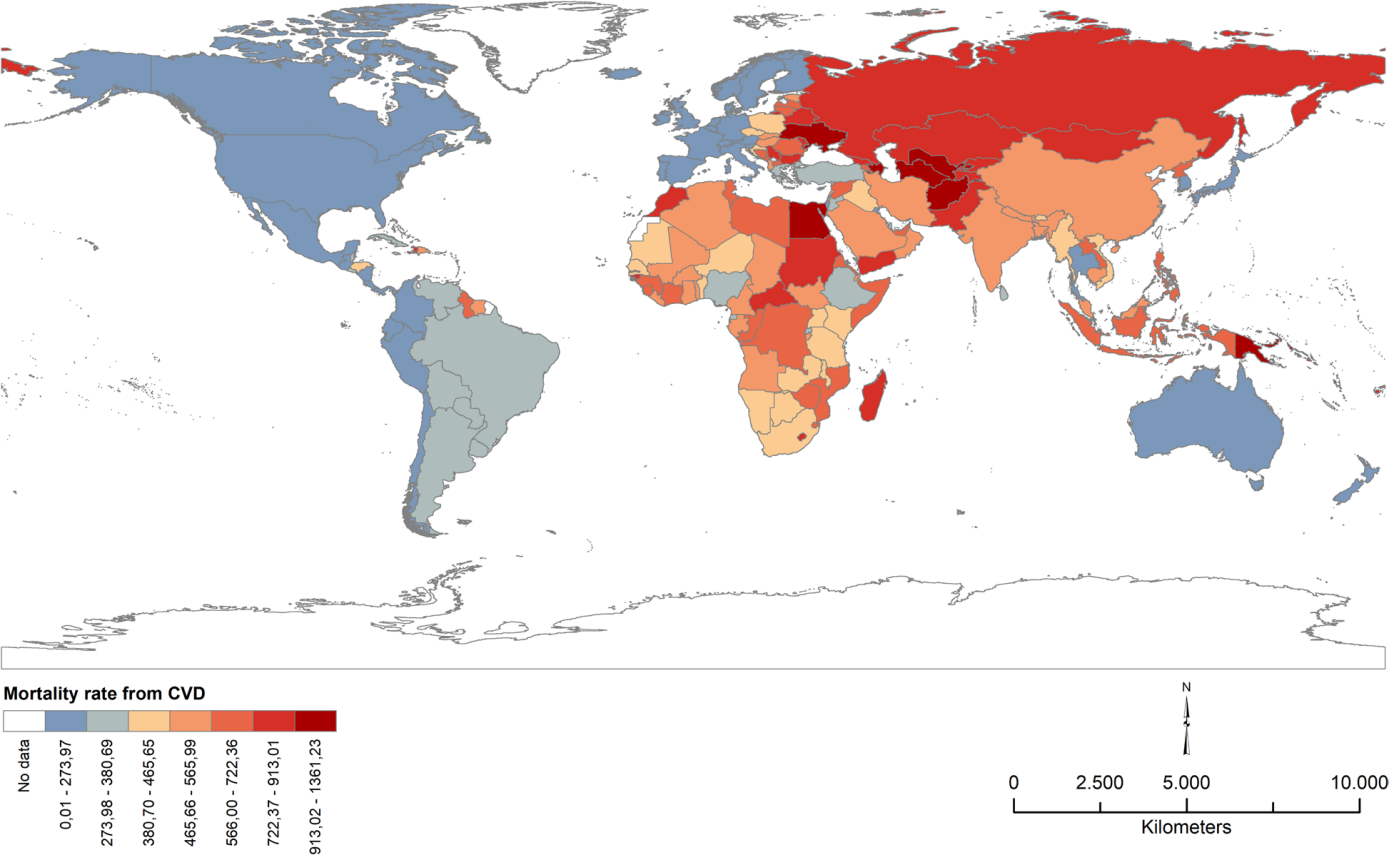
Age-standardized crude mortality rate from cardiovascular disease (per 100,000) by countries – 2013–2017

The spatial distribution of the age-standardized crude mortality rate from cardiovascular disease across the 187 countries under study is shown in Fig. 1. In general, countries in Asia, Africa, and Eastern Europe have higher rates of mortality from CVDs than countries in the Americas (North, Central, and South), Oceania, and other European countries (Northern, Western, and Southern Europe). In this study, Japan is the country with the lowest CMRCVD (142.70), followed by South Korea (154.07), and France (154.51), while at the other extreme are Uzbekistan (1,361.23), Afghanistan (1,154.87), and Papua New Guinea (1,092.59). Mortality rates from cardiovascular disease still have an average of 498.13 per 100,000 population (Table 1).

**Table 1:** Descriptive statistics of dependent and independent variables (*N* = 187)

	Mean	SD	Min	Max	VIF^a^
Mortality rate from cardiovascular disease	498.13	234.84	142.70	1,361.23	NA
GDP per capita	17,392.77	19,116.78	622.00	119,749.00	1.973
% urbanization	57.37	22.71	12.09	100.00	2.023
Schooling	13.10	2.99	4.90	23.30	2.093
Cigarettes	16.99	8.08	3.30	41.10	1.236

^a^Variance inflation factor (VIF) = measure of multicollinearity among the independent variables

Table 1 also summarizes the descriptive statistics of the independent variables in this study. The minimum and maximum gross domestic product per capita (2011 PPP $) found by country was, respectively, $622.00 in Central African Republic and $119,749.00 in Qatar, with average of $17,392.77 and standard deviation of 19,116.78, which indicates that the distribution of GDP per capita varies greatly across countries. Urbanization rates by country range from 12.09% (Burundi) to 100.00% (Kuwait and Singapore), with standard deviation of 22.71, which shows how heterogeneous the distribution is. The average expected years of schooling was 13.10 years ranging from 4.9 years (South Sudan) to 23.3 years (Australia), with standard deviation of 2.99, which suggests that the values are concentrated around the average. Finally, the percentage of men and women, of all ages, who smoke daily range from 3.3% (São Tomé and Príncipe) to 41.10% (Kiribati), with average of 16.99%.

These variables are expected to capture different dimensions of CVD mortality in a country. However, they may have some correlation with each other, which makes it essential to verify if the predictor variables introduce multicollinearity in the analyzes that may compromise our results and conclusions. Therefore, variance inflation factor (VIF) is used to answer this question. Although O’Brien [54] shows “that the rules of thumb associated with VIF (and tolerance) need to be interpreted in the context of other factors that influence the stability of the estimates of the *i*th regression coefficient,” often a VIF value greater than 10 is used to indicate excessive or serious multicollinearity [55–58]. The largest VIF value in our data (Table 1) was 2.093, which is substantially smaller than 10 and therefore provides us evidence that multicollinearity is not a concern in this study.

### Spatial analysis results

Following the proposed analytic strategy, we proceed with the estimation of the three regression models implemented in this study: ordinary least squares (OLS), spatial lag, and spatial error (Table 2). We present the most relevant findings.

**Table 2:** Results of different regression approaches (*N* = 187)

	OLS model	Spatial lag model	Spatial error model
Intercept	808.53 *** (70.086)	693.655 *** (73.485)	944.748 *** (74.649)
GDP per capita	-0.003 *** (0.001)	-0.003 *** (0.001)	-0.004 *** (0.001)
% urbanization	-1.352 (0.867)	-1.567 (0.816)	-1.277 (0.765)
Schooling	-26.400 *** (6.682)	-22.446 *** (6.364)	-30.762 *** (5.765)
Cigarettes	10.110 *** (1.904)	9.065 *** (1.796)	6.146 *** (1.873)
*Model diagnostics*			
R^2^	0.37	0.33^a^	0.34^a^
AIC	2,495.36	2,481.06	2,431.18
Log likelihood	-1,242.68	-1,234.53	-1,210.59
Likelihood Ratio Test (Prob)	―	16.30 (0.000)	64.18 (0.000)
*Spatial effect*			
Rho (spatial lag)	―	0.212 *** (0.051)	―
Lambda (spatial error)	―	―	0.658 *** (0.058)
*Spatial dependence diagnostics*			
Moran’s I	0.498 *** (8.303)	―	―
Lagrange Multiplier	―	16.972 ***	61.449 ***
Robust Lagrange Multiplier	―	0.056	44.533 ***

The Standard Error is presented in parentheses

^a^*pseudo-R*^*2*^

Signif. codes: *p ≤ 0.05; **p ≤ 0.01; ***p ≤ 0.001

First, the three models agree on the algebraic sign (positive or negative) of all coefficient estimates. One should be careful, because the analysis is at the population level and not at the individual level. GDP per capita (2011 PPP $), expected years of schooling, and daily smoking prevalence (cigarettes) were statistically significant (*P* =  < 0.001). The relationship between CMRCVD and the first two is negative, that is, when GDP per capita and schooling increase mortality from cardiovascular disease tends to fall. We observed that countries with higher income and higher educational levels have lower levels of CMRCVD mortality. On the other hand, when cigarette consumption increases CVD mortality also tends to increase, that is, countries with high prevalence of smoking is related to higher mortality. Meanwhile, the percentage of population residing in urban areas, although having a negative relationship with CMRCVD, was not statistically significant (*P* = 0.1204).

Second, the OLS model shows the highest value (2,495.36), and the spatial error model has the lowest AIC value (2,431.18). This result supports the argument that a classic approach (OLS) does not take into account the characteristics of the data and may underestimate the relationships between the explanatory and dependent variables [52, 59].

Another measure that allows comparability between OLS model and spatial regression models is log-likelihood. The higher the log-likelihood, the better the fit. In our case, the spatial error model has greater log-likelihood value (-1,210.59). We can still compare models using the likelihood ratio test ${2}\left[{\text{log}}{\text{L}}_{\text{spatial}}\text{-log}{\text{L}}_{{\text{O}}{\text{LS}}}\right]$. This is a test on the null hypothesis that *ρ* = 0, that is, it is not a test on remaining spatial autocorrelation [45]. When we compare both spatial models with the OLS model for a χ^2^ variate with 1 degree of freedom, we see a significant improvement in fit of the spatial models over the OLS model (likelihood ratio is 16.30 to spatial lag model and 64.18 to spatial error model, *P* =  < 0.001), with a better fit for the spatial error model.

Regarding spatial effects, the spatial autoregressive coefficient (Rho in Table 2) of 0.212 is highly significant. That is, if the average CVD mortality rate of neighboring countries increases by 1%, the CVD mortality rate of a particular country will increase 0.212%. This relationship does not involve other explanatory covariates. As for the coefficient on the spatially correlated errors (Lambda in Table 2) it has a positive effect, and it is highly significant (0.658). This suggests that variables that contribute to CVD mortality rates at the country level may not be included in the analysis.

When compared, we can see that both spatial models yield improvement to the original OLS model, with a better fit for the spatial error model. This leads us to conclude that the control of spatial dependence is an important factor that will improve the performance of our model.

However, and to validate the choice of the better model for our study, another important set of diagnostics that consists of tests of spatial dependence was performed. The first statistic is Moran’s *I*, possibly the most frequently applied test statistic for spatial autocorrelation. We estimate Moran’s *I* for the residuals of the OLS model [60]. The resulting Moran’s *I* score of 0.498 (z-value of 8.303) is highly significant, suggesting strong global spatial autocorrelation of the residuals. This is another indication that the OLS model is not the most suitable for our study, since the same tends to break down in the face of spatial dependence. However, and according to Anselin and Rey [45], when the null hypothesis (no spatial autocorrelation) is rejected by Moran’s *I*, this also does not mean that, necessarily, the alternative of spatial error autocorrelation should be adopted, which is how this result is typically interpreted (incorrectly). They point out that Moran’s *I* also has substantial power against a spatial lag alternative.

In this way, Lagrange Multiplier (LM) test statistics are useful in suggesting which alternative specification should be used. We present four Lagrange Multiplier (LM) test statistics. Lagrange Multiplier (lag) and robust Lagrange Multiplier (lag), as well as Lagrange Multiplier (error) and robust Lagrange Multiplier (error), refer to the spatial lag and spatial error models as the alternatives, respectively. The results show that the statistics of both LM-lag and LM-error are highly significant, rejecting the null hypothesis and indicating the presence of spatial dependence. This leads us to consider the robust tests to help us understand what type of spatial dependence may be at work. The measure for robust LM-error is still significant, but the robust LM-lag test becomes non-significant, which means that when lagged dependent variable is present the error dependence disappears. This suggests that the spatial process generating the data may operate more on the error component of the data than the dependent variable. In summary, these results confirm that the spatial error model, among the tested models, is the one that must be adopted for the study of mortality from cardiovascular diseases.

## Discussion

The paper assesses the relationship between CVD mortality and socioeconomic and cultural variables in the adult population (over 30 years of age) across countries from 2013 to 2017. The aim of this paper is not to examine the causal relationship between CVD mortality and socioeconomic and behavioral factors, but to investigate the global situation of CVD mortality and to raise / explore some research questions about these associations. To do so, we perform a statistical analysis using traditional regression models that incorporate spatial dependence and allow us to investigate the relationship between them.

Mortality by cardiovascular diseases are related to population age structure, prevalence of risk factors, health conditions, institutional factors, the environment, and the socioeconomic situation to which the population is exposed [8, 13]. In other words, there are several determinants and drivers, but that, in most cases, are not uniformly distributed across countries. Below we present further evidence of the relationships studied separated by continents.

Our findings indicate that, in Oceania, with the exception of Australia and New Zealand, the other countries have high CVD mortality rates and low GDP per capita. According to Roth et al. [2], significant declines have been observed in the age-standardized CMRCVD over the past two decades in many middle-income countries, except for several countries in Oceania. Regarding urbanization, Oceania is a peculiarity since most countries are located on islands (island countries). Although some of the smallest nations in this part of the world have the highest rates of urbanization, such as Fiji, Kiribati, and Marshall Islands, most of the countries studied are in the low quintile, with an average urbanization of 22%. The expected years of schooling are mainly in the low and medium quintiles, although in Australia and New Zealand the number of years of schooling that a child of school entrance age can expect to receive is 23.3 (the highest value registered among all the countries studied) and 18.9 (ranked in sixth) years, respectively. On the other hand, most of the countries are located in the highest quintile in relation to the prevalence of daily smoking, being that among the five countries with the highest prevalence, three are in Oceania (Kiribati, Papua New Guinea, and Tonga). These countries had a higher estimated prevalence of daily smoking among women when compared to others in the region, while for men the prevalence was greater than 50% in Kiribati, Papua New Guinea, and Timor-Leste [61].

In Asia, except for cigarettes, the relationship between CVD mortality and the other explanatory variables is quite heterogeneous, a result of their own socioeconomic, behavioral, cultural, spatial, and demographic diversity that characterize the continent. Regarding the relationship between CVD mortality and GDP per capita, of the six countries ranked with the lowest CVD mortality rates, four are in Asia (1st Japan, 2nd South Korea, 4th Israel, and 6th Singapore), all of which are in the high quintile of GDP per capita. At the other extreme, Uzbekistan and Afghanistan, in that order, have the highest CVD mortality rates among all 187 countries studied. In addition, it is worth mentioning the United Arab Emirates, which has one of the highest GDP per capita in the world and is also in the highest quintile of CVD mortality rates. According to Roth et al. [2], significant declines in the age-standardized CMRCVD occurred over the past two decades in many middle-income countries, with the exception of multiple countries in Southeast Asia, as well as Pakistan, Afghanistan, Kyrgyzstan, and Mongolia. Regarding the relationship between CVD mortality and urbanization, the highest percentages of urbanization are located in the Persian Gulf region (Iraq, Israel, Kuwait, Oman, Palestine, Qatar, Saudi Arabia, United Arab Emirates, etc.), East (Japan and South Korea), and Southeast (Brunei, Malaysia, and Singapore) Asian. In these countries, CVD mortality rates are in the low and medium quintiles. In general, in the Persian Gulf countries, oil revenues invested in health and welfare facilities may be an explanation for the reduction in CVD mortality rates [4]. In the second group of countries, policies for obtaining optimal and equitable health for the population are among the main concerns on the public health agenda [62]. The number of years that a child of school entrance age can expect to receive is in the low and medium quintiles in 75% of the Asian countries. According to the Asia Development Bank [63], while much progress has been made in recent years, indicators still point to serious education and human-resource shortfalls across the region. Finally, the prevalence of daily smoking is in the middle and high quintiles in approximately 89% of Asian countries. Ng et al. [61] point out the prevalence of smoking among women in Nepal was comparatively higher than other Asian countries. On the other hand, estimated prevalence was very high among men in South, Southeast, and East Asia.

In Europe, Eastern countries have higher CVD mortality rates than other countries on the continent, although they have declined rapidly over the past twenty-seven years of available data (1990–2017) [2]. Regarding the relationship CVD mortality-GDP per capita, there is a transition in the east–west direction between low mortality / high GDP per capita to high mortality, especially in countries that belonged to the former Soviet Union (USSR), and medium / high GDP per capita. The percentage of urbanization is mainly in the medium / high quintiles. Whether in relation to the urban land expansion or increasing population share, urbanization in Europe is an ongoing phenomenon [64]. The relationship between CVD mortality rates and schooling is almost similar to that observed between the former and GDP per capita. In 80% of countries, the expected years of schooling is at the highest quintile. This says a lot about the many good indicators found in European countries, since education stimulates economic growth and improves people's lives through many ways, including improving health [65]. The prevalence of daily smoking is also observed at the highest quintile for 77% of countries. Greece, Bulgaria, Russia, Cyprus, and Bosnia and Herzegovina are the European countries with the highest prevalence and also those where health risks are most likely to occur [61]. Moldova deserves special mention, as it is the only country on the continent that has high CVD mortality rates and is in the low quintile of GDP per capita, urbanization, and schooling, and high quintile of cigarette use.

In Africa, it is observed that CVD mortality rates are mainly in the medium / high quintiles. Although Africa is still lagging behind in the stage of epidemiological transition, the prevalence of chronic non-communicable diseases (NCDs), such as cardiovascular diseases, have increased in recent years, while the occurrence of communicable diseases have decreased [4]. On the other hand, 75% of African countries are in the low quintile of GDP per capita, which says a lot about how much the continent still has to go in the fight against poverty and inequality. Regarding urbanization, approximately 64% of countries are in the low quintile, which shows that the continent is still largely rural, although it is one of the fastest urbanizing regions in the world [66]. The relationship between CVD mortality rates and schooling is almost similar to that observed between the former and GDP per capita. The number of years that a child of school entrance age can expect to receive is in the low quintile in 71% of the countries studied. Only three countries (Tunisia, Seychelles, and Mauritius) are at the highest quintile, the last two being islands. At the same time that African countries, in general, present poor indicators for the other explanatory variables, the prevalence of daily smoking is in the lowest quintile for 69% of the countries. However, and according to the WHO [67], the prevalence of tobacco smoking appears to be increasing in the African region. Overall, there are fewer studies about CVD mortality in Africa. Mensah et al. [15] investigates cardiovascular mortality trends in Sub-Saharan Africa in the past two decades (1990–2013). They show that CVD mortality represents a small percentage of overall mortality in the region. However, they also suggest that there is a recent increase in CVD mortality related to changes in population age structure and the continuous process of epidemiological transition. In this paper, we find similar trends of CVD mortality in the region and relative high levels of CVD mortality in countries with lower socioeconomic conditions and an increase in smoking (important risk factor). Moreover, the health care system in the region is not mature enough to cover the demands of the population and the region might need to provide a health system for both non-communicable and communicable diseases in the context of changes in population age structure.

In the Americas, CVD mortality is the main cause of death, although there are important regional variations. Recent studies suggest that the number of deaths by CVD will continue to increase in the next few years [68, 69]. Rapid changes in population age structure, high income inequality, urbanization, changes in lifestyle (such as unhealthy diets, increased smoking and obesity and decreased physical activity), and limited access to effective health care are the main causes of the increasing importance of CVDs. According to Roth et al. [2], from 1990 to 2015, Brazil, Canada, and the United States showed a significant decline in the age-standardized CMRCVD. In the United States, Acosta et al. [70] show that CVD mortality is higher than in other countries with similar levels of development (Europe). In recent years, mortality levels have been declining much slower, with much of this stagnation explained by an increase in CVD mortality in working-age population and negative impacts of alcohol use and obesity in the trends of CVD mortality in the US. In addition, Peru had the lowest CVD mortality rates among countries on the continent and was ranked 4th overall. Regarding the relationship between CVD mortality and GDP per capita, there appears to be a clearer division between North, Central, and South. The farther from the equator, the lower is the mortality rate and the higher the GDP per capita. As for urbanization, in 2014 Central and South America became the most urbanized regions in the world, where 80% of the population lived in cities [71]. The expected years of schooling in 57% of the countries are in the medium quintile. Urquiola and Calderón [72] state that if the overall enrollment rates are considered, it shows that Latin America countries spend substantial resources on education. Finally, the prevalence of daily smoking is in the lowest quintile for 63% of the countries. However, there are large variations within the region. Bolivia, Chile, and Uruguay are the only countries that have CVD mortality rates and prevalence of daily smoking in the low and high quintiles, respectively. For women, Chile and Uruguay have much higher estimated prevalence rates than other countries in the region [61]. Regional health-system planning needs an understanding of the absolute burden of cardiovascular disease and the effect of demographic and economic changes. Regions with a declining incidence of cardiovascular diseases may still need to invest heavily in health promotion and treatment given trends in population age structure that might increase the number of deaths from this specific cause. In addition, some countries in Latin America might need to invest heavily in the healthcare system and preventable policies to reduce the possible impacts of CVD mortality [73, 74].

In summary, regional disparities in the distribution of health-disease patterns among countries are very important [13, 75]. In most low- and middle-income countries there is still high prevalence of communicable diseases (diarrhea, lower respiratory, HIV / AIDS, tuberculosis and other common infectious diseases), while in more developed economies and those at an advanced stage of the epidemiologic transition process, the prevalence of chronic non-communicable diseases (NCDs) is observed, in particular, cardiovascular diseases.

We argue that this study provides useful clues for policymakers establishing effective public health planning and measures for the prevention of deaths from cardiovascular disease. The reduction of CVD mortality can positively impact GDP growth because increasing life expectancy enables people to contribute to the economy of the country and its regions for longer. Some important research issues raised by this paper should be considered in future studies. The relationship between CVD and income, and other socioeconomic variables, are important. In addition, it is important to understand how changes in individual behavior across different countries might affect future trends in CVD mortality, especially related to smoking and dietary behaviors. Some studies show a reduction in the decline in CVD mortality in high-income countries [8]. However, in less developed regions of the world, we observe increasing levels of CVD mortality and still relatively low levels of economic development. Thus, future research should focus on the trends of less developed economies in terms of health behavior and mortality trends. How are health measures dealing with the aging process and changes in population behavior? Finally, it is important to consider how public and private health care systems are organized and organizing themselves to deal with these changes across the globe. These questions need to be on the research agenda.

However, this research is also subject to limitations. First, our study is at the aggregated level and there might be important variations within countries and among individuals. As shown before in other studies, some regions of very large countries can behave quite differently on the relation between development and cardiovascular mortality [26, 76, 77]. In the case of CVD mortality, further research should investigate individual behavior and its relation to the macro environment to obtain further knowledge on proper interventions to reduce the levels of mortality. Our study is also limited by the availability of explanatory variables that help to understand levels and trends of CVD mortality across countries. We were limited that variations that are available for all countries and, in some cases, they also present their limitations. Finally, a large percentage of countries do not have a proper operational civil registration and vital statistic (CRVS) system, thus mortality and causes of deaths are based on analytical models using data from regions with adequate data. This reinforces the importance to build and invest in CRVS systems across the globe [78].

## Conclusion

Although we show that there is large variation in CVD mortality levels across countries in recent years, we observe an increase in CVD mortality in less developed countries and a stagnation in the decline of CVD in more developed economies. We produce a comparative analysis of CVD mortality and GDP per capita, urbanization, schooling and cigarettes across countries and found how each variable relates to the level of mortality. The relationship between CVD and socioeconomic variables is important, as well as understanding how changes in individual behavior across different countries might affect future trends in CVD mortality, especially related to smoking and dietary behaviors.

## Supplementary Information

**Additional file 1.** 

## Data Availability

The datasets generated and/or analysed during the current study are not publicly available due to file size, but are available from the corresponding author on reasonable request.

## Abbreviations

AIC: Akaike Information Criterion
CMRCVD: Crude mortality rate from cardiovascular disease
CRVS: Civil registration and vital statistic
CVD: Cardiovascular disease
EDA: Exploratory data analysis
GBD: Global Burden of Disease
GDP: Gross domestic product
IHME: Institute for Health Metrics and Evaluation
NCD: Non-communicable disease
OLS: Ordinary least squares
PPP: Purchasing power parity
SGD: Sustainable Development Goal
UNDESA: United Nations Department of Economic and Social Affairs
UNDP: United Nations Development Programme
VIF: Variance inflation factor
WHO: World Health Organization
