# Supplementary material for: Spatial analysis of cardiovascular mortality and associated factors around the world
Source: BMC Public Health. 2022 Aug 16;22:1556. doi: 10.1186/s12889-022-13955-7 (PMC9380346; doi:10.1186/s12889-022-13955-7)
Supplement: Supplementary file 1 — Additional file 1. [file 12889_2022_13955_MOESM1_ESM.docx]

**Additional file 1**

| **Africa** | **America** | **Asia** | **Europe** | **Oceania** |
| --- | --- | --- | --- | --- |
| Algeria | Antigua and Barbuda | Afghanistan | Albania | Australia |
| Angola | Argentina | Azerbaijan | Andorra | Fiji |
| Benin | Bahamas | Bahrain | Armenia | Kiribati |
| Burkina Faso | Barbados | Bangladesh | Austria | Macedonia |
| Burundi | Belize | Bhutan | Belarus | Marshall Islands |
| Cameroon | Bolivia | Brunei | Belgium | Micronesia |
| Cape Verde | Brazil | Cambodia | Bosnia and Herzegovina | New Zealand |
| Central African Republic | Canada | China | Botswana | Papua New Guinea |
| Chad | Chile | India | Bulgaria | Samoa |
| Comoros | Colombia | Indonesia | Croatia | Solomon Islands |
| Côte d'Ivoire | Costa Rica | Iran | Cyprus | Timor-Leste |
| Democratic Republic of the Congo | Cuba | Iraq | Czech Republic | Tonga |
| Djibouti | Dominica | Israel | Denmark | Vanuatu |
| Egypt | Dominican Republic | Japan | Estonia |  |
| Eritrea | Ecuador | Jordan | Finland |  |
| Ethiopia | El Salvador | Kazakhstan | France |  |
| Gabon | Equatorial Guinea | Kuwait | Georgia |  |
| Gambia | Grenada | Kyrgyzstan | Germany |  |
| Ghana | Guatemala | Laos | Greece |  |
| Guinea | Guyana | Lebanon | Hungary |  |
| Guinea-Bissau | Haiti | Malaysia | Iceland |  |
| Kenya | Honduras | Maldives | Ireland |  |
| Lesotho | Jamaica | Mongolia | Italy |  |
| Liberia | Mexico | Myanmar | Latvia |  |
| Libya | Nicaragua | Nepal | Lithuania |  |
| Madagascar | Panama | North Korea | Luxembourg |  |
| Malawi | Paraguay | Oman | Malta |  |
| Mali | Peru | Pakistan | Moldova |  |
| Mauritania | Saint Lucia | Palestina | Montenegro |  |
| Mauritius | Saint Vincent and the Grenadines | Philippines | Netherlands |  |
| Morocco | Suriname | Qatar | Norway |  |
| Mozambique | Trinidad and Tobago | Saudi Arabia | Poland |  |
| Namibia | United States | Singapore | Portugal |  |
| Niger | Uruguay | South Korea | Romania |  |
| Nigeria | Venezuela | Sri Lanka | Russia |  |
| Republic of Congo |  | Syria | Serbia |  |
| Rwanda |  | Tajikistan | Slovakia |  |
| São Tomé and Príncipe |  | Tanzania | Slovenia |  |
| Senegal |  | Thailand | Spain |  |
| Seychelles |  | Turkey | Sweden |  |
| Sierra Leone |  | Turkmenistan | Switzerland |  |
| Somalia |  | United Arab Emirates | Ukraine |  |
| South Africa |  | Uzbekistan | United Kingdom |  |
| South Sudan |  | Vietnam |  |  |
| Sudan |  |  |  |  |
| Swaziland |  |  |  |  |
| Togo |  |  |  |  |
| Tunisia |  |  |  |  |
| Uganda |  |  |  |  |
| Yemen |  |  |  |  |
| Zambia |  |  |  |  |
| Zimbabwe |  |  |  |  |
